# Impact of sperm DNA fragmentation on the clinical outcome of assisted reproduction techniques: a systematic review of the last five years

**DOI:** 10.5935/1518-0557.20220057

**Published:** 2023

**Authors:** Mayara Lobato Lourenço, Gabriel Acácio de Moura, Yasmim Mendes Rocha, João Pedro Viana Rodrigues, Paula Bruno Monteiro

**Affiliations:** 1 Evangelista Torquato Human Reproduction Clinic, Fortaleza-CE, Brazil; 2 Oswaldo Cruz Foundation (FIOCRUZ), Eusébio-CE, Brazil; 3 Conceptus Human Reproduction Clinic, Fortaleza-CE, Brazil

**Keywords:** fragmentation of DNA sperm, male infertility, *in vitro* fertilization, Intracytoplasmic sperm injection, intrauterine insemination

## Abstract

**Objective:**

To elucidate through a systematic literature review the impact sperm DNA fragmentation has on embryos from assisted reproduction techniques.

**Data Source:**

Studies from the “PubMed”, “Embase”, and “BVS” databases were analyzed.

**Studies Selection:**

The articles selected in the review included: cohort and case-control studies that addressed the proposed theme, published between January 1, 2017, and January 31, 2022, in English, Portuguese, and Spanish. As inclusion criteria: cohort and case-control articles. As exclusion criteria: articles outside the scope of the research, review articles, case reports, articles using animal models, abstracts, letters to the editor, and articles found duplicates in the databases.

**Data Collection:**

Number of couples or cycles; age (men/women); collection type; DNA damage (%); assisted reproduction activity and techniques.

**Data Synthesis:**

*In in vitro* fertilization, a reduction in fertilization rate, blastocyst rate, and embryo quality was observed. In addition to implantation and increased abortion rates in patients with high sperm DNA fragmentation. High rates of sperm DNA fragmentation in intracytoplasmic sperm injection led to reduced blastocyst production rate, embryo quality, implantation, and live birth rate, and in intrauterine insemination, a reduction in pregnancy rate.

**Conclusion:**

Sperm DNA fragmentation was a potential limiting factor for assisted reproduction techniques.

## INTRODUCTION

Currently, semen analysis is still a laboratory technique considered the gold standard for attempting to identify male infertility (Douglas et al., 2021). This technique is based on the principle that infertility can often be triggered by several factors that alter parameters, including sperm motility, morphology, liquefaction time, seminal volume, sperm concentration, and sperm motility (Yu et al., 2018). Other tools for evaluating semen have allowed us to explore these parameters that refer to unexplained male fertility, such as the evaluation of anti-sperm antibodies, sperm hyperactivation, acrosomal reaction, penetration in the zona pellucida, and Sperm DNA fragmentation (SDF) (Esteves et al., 2014).

An important fact is that even with the advent of Assisted Reproductive Techniques (ART), the paternal factor can significantly interfere with the pregnancy obtained. Furthermore, it has been suggested that embryo quality parameters may be directly correlated with seminal quality (Martínez et al., 2021). This phenomenon may be associated with delivering damaged genetic material from the spermatozoon to the oocyte (Li & Lloyd, 2020). Notwithstanding this, it is becoming increasingly common for the integrity of our genome to be continuously challenged by endogenous products and exogenous factors (Santi et al., 2018). These products can endogenously damage spermatogenesis during meiosis by replacing histones with protamines. They can also be triggered by accumulated DNA damage during maturation and storage in the epididymis (Cissen et al., 2016).

Given all the above, new methodologies for enhancing semen arise to optimize the functional analysis of sperm (Borges Jr. et al., 2019). Among them, we can mention the terminal deoxynucleotidyl transferase (TUNEL) mediated dUTP endpoint assay, sperm chromatin dispersion (SCD) test, the comet assay, and the sperm chromatin structure assay (SCSA) (Santi et al., 2018). Each test is known to provide different forms of DNA damage. It is also already accepted that these assays for identifying SDFs perform a more accurate prediction of male fertility because they have less biological variability than conventional semen analyses (Tan et al., 2019).

A worrying factor is that about 30% of patients seeking ARTs services have high rates of sperm DNA breaks (Bungum et al., 2006). Furthermore, although sperm with fragmented DNA can fertilize an oocyte with similar success to non-fragmented sperm fertilization, the negative impact of damaged paternal chromatin usually manifests as a compromise in its development and subsequent implantation (Esteves et al., 2014). Due to this, the recognition of the effects of SDFs in embryos produced by ARTs such as In Vitro Fertilization (IVF), Intracytoplasmic Sperm Injection (ICSI), and Intrauterine Insemination (IUI) can help to improve the techniques used to identify SDFs as to verify their real impact in procedures (Santi et al., 2018). Therefore, the present work aims to elucidate through a systematic literature review the impact SDFs exert on embryos originating from ARTs.

## MATERIALS AND METHODS

### Types of Study

A systematic review of the literature in the present work was performed using the databases of Pubmed, Embase, and Virtual Health Library (VHL). All work was performed according to the rules guide Reporting Items for Systematic Reviews and Meta-Analysis (PRISMA). The entire protocol of the present review is reported in detail in the database of the National Institute of Health Research (PROSPERO) database under protocol number (CRD42021256984).

### Eligibility Criteria for Study Selection

As study eligibility criteria, cohort and case-control articles that addressed the authors’ proposed topic were selected. To minimize bias rates with respect to advances and improvements in assisted reproduction techniques over time, we adopted five years from January 1, 2017, to January 31, 2022, in English, Portuguese and Spanish. As the main intervention methods analyzed, we selected articles that used couples with male partners with high or low rates of SDF and resorted to ARTs. Furthermore, as clinical outcomes, we observed the rate of fertilization, miscarriage, live births, chromosomal changes, and the dynamics of embryonic development and methods of detecting SDF. All eligibility criteria are shown in Table 1.

**Table 1:** Systematic search using the PICO tool.

Description	Abbreviation	Component Questions
Population	P	Couples who resorted to the use of assisted reproduction techniques with the male partner having a low or high rate of sperm DNA fragmentation
Intervention	I	Assisted reproductive treatments (*in vitro* fertilization, intracytoplasmic sperm injection, and artificial insemination)
Comparison	C	Couples with unchanged fertility (when applicable)
Outcome	O	Rate of fertilization, abortion, live births, chromosomal changes, and the dynamics of embryonic development
Studies	S	Cohort and case-control studies

### Methodological analysis of the studies

After the search and selection, an internal quality analysis was carried out among the studies by the authors. Based on the methodological designs used in each article, the Newcastle Ottawa scale was used for cohort and case-control studies to reduce the risk of bias among the studies selected for this review. The methodological analysis criteria of the studies are presented in Table 2.

**Table 2:** Newcastle-Ottawa Scale to evaluate the internal quality of studies.

Study	NOS Items Score								
**Criteria**	**Selection 1**	**Selection 2**	**Selection 3**	**Selection 4**	**Comparability 1a**	**Results 1**	**Results 2**	**Results 3**	**Total**
Pabuccu *et al*., 2017	0	1	1	1	0	1	1	1	6
Gat *et al*., 2018	0	1	1	1	0	1	1	1	6
Sun *et al*., 2018	1	1	1	1	0	1	1	1	7
Tello-Mora *et al*., 2018	1	1	1	1	0	1	1	1	7
Zhang *et al*., 2019	1	1	1	1	0	1	1	1	7
Salehi *et al*., 2019	0	1	1	1	0	1	1	1	6
Casanovas *et al*., 2019	0	1	0	1	0	1	1	1	5
Antonouli *et al*., 2019	1	1	1	1	0	1	1	1	7
Bichara *et al*., 2019	1	1	1	1	1	1	1	1	8
Zarén *et al*., 2019	1	1	1	1	0	1	1	1	7
Green *et al*., 2020	1	1	1	1	0	1	1	1	7
Alharbi *et al*., 2020	0	1	1	1	0	1	1	1	6
Cheng *et al*., 2020	1	1	1	1	0	1	1	1	7
Anbari *et al*., 2020	0	1	1	1	0	1	1	1	6
Kabukçu *et al*., 2021	0	1	1	1	0	1	1	1	6
Setti *et al*., 2021	1	1	1	1	0	1	1	1	7
Rex et al., 2021	1	1	1	1	0	1	1	1	7
Tang *et al*., 2021	1	1	1	1	1	1	1	1	8
Vončina *et al*., 2021	0	1	1	1	0	1	1	1	6
Wang *et al*., 2022	1	1	1	1	0	1	1	1	7

Selection 1: Representativeness of the exposed cohort; Selection 2: Selection of the unexposed cohort; Selection 3: Exposure determination; Selection 4: Demonstration that the result of interest was not present in the baseline; Comparability 1a and 1b: Comparability of cohorts based on design or analysis; Results 1: Results evaluation; Results 2: Follow-up of cohorts; Results 3: Adequacy of cohort follow-up.

### Inclusion and Exclusion Criteria

As selection criteria for the preparation of the present study, inclusion and exclusion criteria of articles were determined. The inclusion criteria were cohort and case-control articles. Exclusion criteria were articles outside the scope of the study, review articles, case reports, articles using animal models, abstracts, letters to the editor, and articles found duplicates in the databases.

### Data Collection and Extraction

A database search was performed using the descriptors. Subsequently, two authors evaluated the titles and abstracts of the articles (GLLM; MGA). After analysis, these were tabulated. When there was a divergence, these results were reanalyzed by an expert author in the area (PBM). The data extracted from the studies were:

Number of couples or cycles;

- Age (men/women);
- Type of collection;
- Methodology for SDF detection;
- DNA damage (%);
- Outcomes; and
- ART.

## RESULTS

**Figure 1:**
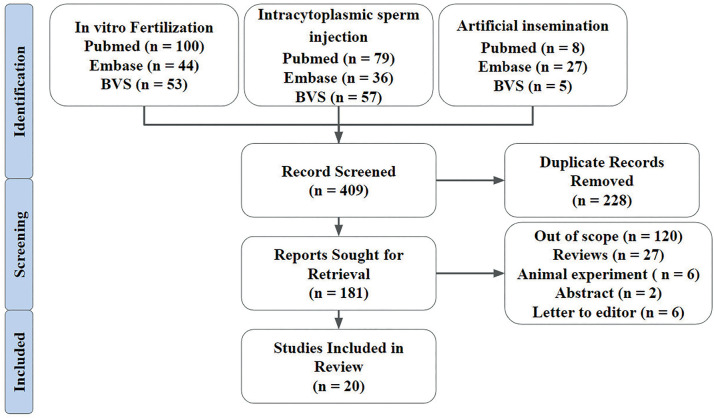
Methodological flowchart.

According to the methodological parameters used in the present review, 409 articles were found, of which only 20 were selected according to the adopted inclusion and exclusion criteria. The entire search and screening protocol is briefly described in Figure 1. In total, 8123 couples and 95 cycles were evaluated in the three ARTs addressed in patients with a mean age ranging from 29.20 to 43.60 years in male partners and 21 to 38.26 years in female patients. The selected articles used flow cytometry, TUNEL, SCD, and SCSA. All data are described in Tables 3, 4, and 5.

**Table 3:** SDF activity in the clinical outcome of the *in vitro* fertilization biotechnique.

Author	Number of Couples or Cycles	Age (Men)	Age (Women)	Type of Seminal Collection	Methodology for SDF detection	(%) Sperm DNA Damage	Clinical Outcome
Gat *et al.*, 2018	45 cycles	Average age: ↓ SDF (43.6 years) ↑ SDF (40.6 years)	Average age: ↓ SDF (25.6 years) ↑ SDF (25.7 years)	-	Flow Cytometry	↓ SDF (<15%) ↑ SDF (>15%)	No significant difference
Sun *et al*., 2018	238 couples	Average age: ↓ SDF (37.72 years) ↑ SDF (40.75 years)	Average age: ↓ SDF (37.50 years) ↑ SDF (38.26 years)	-	SCD	↓ SDF (<30%) ↑ SDF (>30%)	No significant difference
Tello-Mora *et al.*, 2018	69 years	Average age: 40 years	-	Masturbation	SCSA	Average 21.7%	↓ Blastocyst Rate
Zarén *et al*., 2019	204 couples	-	25-39 years	Masturbation	SCSA	-	↑ Poor Quality Embryos
Cheng *et al*., 2020	3000 couples (FIV+ICSI+IIU)	Average age: DFI < 15% (31.28 years); 15%< DFI < 30% (32.79 years); DFI > 30% (32.98 years).	Average age: DFI < 15% (29.45 years); 15%< DFI < 30% (28.37 years); DFI > 30% (29.37 years).	-	Flow Cytometry	DFI < 15%; 15%< DFI < 30% DFI > 30%	From 15% (↓ Pregnancy)
Anbari *et al*., 2020	50 couples	-	Average age: 30.76 years	Masturbation	SCD	5-27%	First Cleavage Time Modification; Embryonic Multinucleation; ↑ Fragmentation and Vacuole
Tang *et al*., 2021	116 couples	Average age 33.2 years	Average age: 30.7 years	-	SCD	Average 22.9%	↓ Fertilization Rate
Vončina *et al*., 2021	1224 couples	-	Average age: 32.8 years	-	SCSA	Average 12.9%	↓ Fertilization Rate ↓ Live Birth Rate
Wang *et al.*, 2022	251 couples	-	Average age: ↓ SDF (28.40 years) ↑ SDF (28.90 years)	Masturbation	SCD	↓ SDF (<25%) ↑ SDF (>25%)	↑ SDF: ↓ Fertilization Rate

SCD: Sperm Chromatin Dispersion Assay; SCSA: Sperm Chromatin Structure Assay; SDF: Fragmentation of Sperm DNA; DFI: DNA Fragmentation Index; IVF: *In vitro* Fertilization; ICSI: Intracytoplasmic Sperm Injection; AI: Artificial Insemination; (-): Not Available.

**Table 4:** SDF activity in the clinical trial of the intracytoplasmic sperm injection biotechnique.

Author	Number of Couples or Cycles	Age (Men)	Age (Woman)	Type of Seminal Collection	Methodology for SDF detection	(%) Sperm DNA Damage	Clinical Outcome
Pabuccu *et al*., 2017	92 couples	-	Group: Testicular ICSI (33 years) Ejaculated ICSI (33.9 years)	Testicular Aspiration; Masturbation	TUNEL	Testicular ICSI (44.8%) Ejaculated ICSI (41.7%)	Testicular Aspiration (↑ Embryonic Transference)
Sun *et al*., 2018	152 couples	Average age: ↓ SDF (36.46 years) ↑ SDF (36.44 years)	Average age: ↓ SDF (34.37 years) ↑ SDF (33.87 years)	-	SCD	↓ SDF (<30%) ↑ SDF (>30%)	No significant difference
Zhang *et al*., 2019	102 couples	Group: Testicular ICSI (33.5 years) Ejaculated ICSI (34.6 years)	Group: Testicular ICSI (30.4 years) Ejaculated ICSI (30.1 years)	Testicular Aspiration; Masturbation	SCSA	>30%	Testicular Aspiration (↑ Embryonic Transference)
Salehi *et al*., 2019	50 cycles	-	Average age: ↓ SDF (28.30 years) ↑ SDF (27.10 years)	Masturbation	SCD	↓ SDF (<30%) ↑ SDF (>30%)	↓ Embryonic Quality
Casanovas *et al*., 2019	43 couples	Average age: ↓ Single Strand Damage (37.36 years) ↑ Single Strand Damage (39.12 years) ↓ Double Strand Damage (38 years) ↑ Double Strand Damage (38.17 years)	Average age: ↓ Single Strand Damage (37.27 years) ↑ Single Strand Damage (37.31 years) ↓ Double Strand Damage (37.27 years) ↑ Double Strand Damage (37 years)	-	Comet Assay	-	Delay in Embryonic Kinetics
Antonouli *et al*., 2019	150 couples	43.4 years	-	Masturbation	SCD	25%	No significant difference
Bichara *et al*., 2019	132 couples	33.7 years	34.8 years	-	TUNEL	-	No significant difference
Zarén *et al*., 2019	148 couples	-	21-54 years	Masturbation	SCSA	-	No significant difference
Green *et al*., 2020	234 couples	Average age: ↓ SDF (38.30 years) ↑ SDF (40.50 years)	Average age: ↓ SDF (37.60 years) ↑ SDF (37.80 years)	-	SCSA	↓ SDF (<15%) ↑ SDF (>15%)	No significant difference
Alharbi *et al*., 2020	52 couples	Group: Testicular ICSI (38.9 years) Ejaculated ICSI (37 years)	Group: Testicular ICSI (34.4 years) Ejaculated ICSI (33.5 years)	Testicular Aspiration; Masturbation	SCSA	37.6 %	Testicular Aspiration (↑ Embryonic Transference)
Cheng *et al*., 2020	3000 couples (FIV+ICSI+IIU)	Average age: DFI < 15% (31.28 years); 15%< DFI < 30% (32.79 years); DFI > 30% (32.98 years).	Average age: DFI < 15% (29.45 years); 15%< DFI < 30% (28.37 years); Average Age: DFI > 30% (29.37 years).	-	Flow Cytometry	DFI < 15%; 15%< DFI < 30% DFI > 30%	From 15% (↓ Pregnancy)
Setti *et al*., 2021	540 couples	Group: > 36 years 37- 40 years > 40 years	Group: > 36 years 37- 40 years > 40 years	Masturbation	SCD	↓ SDF (<30%) ↑ SDF (>30%)	↑ SDF (>30%): ↓ Embryonic Quality; ↓ Blastocytes Rates; ↓ Pregnancy Rate; ↓ Deployment Rate; Abortion rate.
Vončina *et al*., 2021	995 couples	-	32.8 years	-	SCSA	19.7%	No significant difference

SCD: Sperm Chromatin Dispersion Assay; SCSA: Sperm Chromatin Structure Assay; SDF: Fragmentation of Sperm DNA; DFI: DNA Fragmentation Index; IVF: *In vitro* Fertilization; ICSI: Intracytoplasmic Sperm Injection; AI: Artificial Insemination; (-): Not Available.

**Table 5:** SDF activity in the clinical outcome of artificial insemination biotechniques.

Author	Number of Couples or Cycles	Age (Men)	Age (Women)	Type of Seminal Collection	Methodology for SDF detection	(%) Sperm DNA Damage	Clinical Outcome
Cheng *et al*., 2020	3000 couples (FIV+ICSI+IA)	Average age: DFI < 15% (31.28 years); 15%< DFI < 30% (32.79 years); DFI > 30% (32.98 years).	Average age: DFI < 15% (29.45 years); 15%< DFI < 30% (28.37 years); Average age: DFI > 30% (29.37 years).	Masturbation	Flow Cytometry	DFI < 15%; 15%< DFI < 30% DFI > 30%	From 15% (↓ pregnancy)
Kabuçu *et al*., 2020	120 couples	Average age: Sexual Abstinence 1 day (29.20 years) Sexual Abstinence 2 days (30.20 years)	Average age: Sexual Abstinence 1 day (33.30 years) Sexual Abstinence 2 days (30.20 years)	-	TUNEL	Sexual Abstinence 1 day (20.70%) Sexual Abstinence 2 days (23.73%)	No significant difference
Rex *et al*., 2021	211 couples	-	31.4 years	Masturbation	SCSA	<10%	↓ Pregnancy

SCD: Sperm Chromatin Dispersion Assay; SCSA: Sperm Chromatin Structure Assay; SDF: Fragmentation of Sperm DNA; DFI: DNA Fragmentation Index; IVF: *In vitro* Fertilization; ICSI: Intracytoplasmic Sperm Injection; AI: Artificial Insemination; (-): Not Available.

### The activity of SDFs in the clinical outcome of IVF

The literature has shown that SDF can impair the clinical outcome of patients undergoing IVF protocols. In three articles, the fertilization rate of oocytes by sperm with fragmented DNA was reduced (Tang et al., 2021; Vončina et al., 2021; Wang et al., 2022). In a single study, the blastocyst production rate was also reduced (Tello-Mora et al., 2018). Evidence showed that SDF could lead to altered developmental kinetics and consequently reduced quality of the produced embryo (Zarén et al., 2019; Anbari et al., 2020). In another study, it was found that with only 15% fragmented sperm, there was a drop in the clinical pregnancy rates achieved by these patients (Cheng et al., 2020). The live birth rate was also reduced in these patients (Vončina et al., 2021).

### Action of SDFs on the clinical outcome of patients undergoing ICSI

For couples undergoing ICSI protocols, direct effects on their clinical outcome were verified as in IVF. In one of the studies, 30% of fragmented DNA in the semen sample reduced blastocyst production rates (Setti et al., 2021). In two studies, embryo quality was also altered in ICSI protocols (Salehi et al., 2019; Setti et al., 2021). In only one article, there was a delay in embryo development kinetics (Casanovas et al., 2019). The implantation rate and clinical pregnancy achievement of the patients were also reduced in two studies (Cheng et al., 2020; Setti et al., 2021). Increased abortion rates were observed in one study (Setti et al., 2021). Interestingly, the number of blastocysts and embryo transfer was higher in couples who performed semen collection by Testicular Aspiration (TESA) compared to those who performed masturbation (Pabuccu et al., 2017; Zhang et al., 2019; Alharbi et al., 2020).

### SDF can modulate the clinical outcome of patients undergoing intrauterine insemination

Our search strategy identified a few studies concerning IIU. Among the three articles that addressed intrauterine insemination (IIU), two reported a possible activity of SDFs in obtaining clinical pregnancy by couples undergoing this type of protocol (Cheng et al., 2020; Rex et al., 2021). In one of these studies, the 10% rate of sperm DNA fragmentation was already enough to lead to this reduction.

## DISCUSSION

Infertility is a comorbidity that affects more than 180 million people worldwide, with the male factor found in 10% of all couples and responsible for 50% of cases of infertility (Esteves et al., 2021). SDFs are extremely common in these infertile patients. They have been associated with several critical etiological factors, such as errors in spermiogenesis, impaired chromatin compaction, sperm apoptosis, endogenous caspases and endonucleases, oxidative stress, chemotherapeutic agents, radiation, infection, and lifestyle (Li & Lloyd, 2020). Therefore, exploring the impact of SDF on male fertility and their chances on the clinical outcome of patients who resort to ARTs may be a crucial task, aiming to improve predictive factors to aid clinical outcomes in these patients (Omran et al., 2021).

In our review, we sought to understand the key clinical outcomes of patients undergoing IVF, ICSI, or IUI who had rates of sperm DNA fragmentation. One of our exciting findings was that the presence of sperm with fragmented DNA in IVF reduced the fertilization rate (Tang et al., 2021; Vončina et al., 2021; Wang et al., 2022). Such results were also found in the study by Borini et al. (2006), who found a slight positive correlation between the SDF rate and the oocyte fertilization rate. On the contrary, the literature already has study that do not correlate oocyte fertilization rate with SDF in IVF (Xue et al., 2016). This fact may be associated with an inversely proportional correlation between the SDF parameter and conventional seminal parameters such as concentration, motility, and morphology, that is, patients who have high SDF may have fewer spermatozoa capable of properly fertilizing the oocyte (Evgeni et al., 2015).

It has also been found that in IVF protocols there were problems regarding blastocyst formation, embryo development kinetics, and embryo quality (Tello-Mora et al., 2018; Zarén et al., 2019; Anbari et al., 2020). These results regarding embryo quality were also seen in the study by Oleszczuk et al. (2016), who found that during IVF, from a 20% SDF rate, there is an increased chance of obtaining embryos with inferior quality. Evidence on the delay in the kinetics of embryonic development kinetics has also been shown in a study conducted in mice that verified a delay in DNA replication that caused a substantial delay in progression to the 2-cell stage and entry of inertia at the G2/M stage (Gawecka et al., 2013). Moreover, this fact may be associated with the repair of the genetic material during embryonic development, which the study of Yamauchi et al. (2013) found that in mice, the damage to the genetic material of the sperm persists after fertilization, and to repair this error, the oocyte tries to recover it, thus delaying its development kinetics, and therefore may act directly on the embryo quality.

Other studies have already associated SDF rates with gestational loss and implantation failures (Simon et al., 2013; Coughlan et al., 2015; Muratori et al., 2016). Such results confirm the data observed in our review (Cheng et al., 2020; Setti et al., 2021; Vončina et al., 2021). It is already known that a truncated package protects the sperm chromatin during transport through the male and female reproductive tracts, ensuring the transfer of the intact paternal genome to the oocyte. In mammals, the quality of genome packaging is associated with the number of cysteines in protamine levels, i.e., the higher the number of sulfide bridges, the greater the stability of DNA (Esteves et al., 2021). However, when this process of genomic packaging is defective, an abnormal chromatin structure is created that prevents the zygote from accessing the proper sequences of the paternal genome in embryonic development, which can lead to nonfertilization and gestational loss (Esteves et al., 2021; Esquerré-Lamare et al., 2018).

Our review showed a reduction in blastocyst rates and embryo quality, as well as changes in embryonic kinetics in ICSI procedures in couples with increased DFS (Salehi et al., 2019; Casanovas et al., 2019; Setti et al., 2021). These results agree with the literature, as in Sivanarayana et al. (2014), who found that patients undergoing ICSI with fragmentation >30% had lower blastocysts produced than the control group. It is suggested that this activity may be due to failures in oocyte activation factors. This effect can often be observed from the 4-cell stage with activation of the embryonic genome. However, RNA synthesis can be detected in pronuclei humans, and this transcription failure is directly associated with this abnormal embryonic development (Tesarik et al., 2004).

Another exciting factor observed in our review was the increase in TESA transfer rates concerning ejaculation in SDF patients undergoing ICSI (Pabuccu et al., 2017; Zhang et al., 2019; Alharbi et al., 2020). The literature has verified that TESA and masturbation procedures have similar success rates in ARTs (Al-Malki et al., 2017). TESA is a technique for isolating sperm or spermatids obtained directly from seminiferous tubules from a tissue shear process for use in a procedure (Javed et al., 2019). The central hypothesis confirming this finding is that the DNA damage in testicular sperm is mostly from direct meiotic failure or defective early chromatin in the spermatid stage. In contrast, most DNA damage can be found in the post-testicular region, which is most likely to cause damage to its genetic material (Esteves et al., 2015).

In our search, we obtained few results regarding the impact of SDFs on IUI rates. Among these results, a reduction in pregnancy rates of patients undergoing this procedure was reported (Cheng et al., 2020; Rex et al., 2021). A previous study found that SDF detection was a good predictor of clinical pregnancy in patients undergoing (IUI) (Duran et al., 2002). However, in another study, SDFs showed no significant difference in achieving clinical pregnancy obtained by IUI (Siddhartha et al., 2019). This fact may also be associated with the relationship between SDFs and seminal parameters, which consequently affects the pregnancy rate in these patients (Evgeni et al., 2015).

The present review presents some limiting factors, as in most studies, heterogeneity exists between them. In some studies, another essential factor was the absence of patients as healthy controls. Choosing the five-year period mentioned above in the section on materials and methods limited the number of articles obtained. However, it allowed us to review the most current data in the literature.

In conclusion, the SDF increase proved to be a limiting potential for ARTs. In IVF, clinical outcomes such as reduced fertilization rate, blastocyst rate, embryo quality, reduced implantation rate, and increased abortion rates were observed. In ICSI, outcomes such as reduced blastocyst production rate, embryo quality, implantation, and live birth rate were verified. Furthermore, in IUI, results of reduced pregnancy rates were observed. However, the mechanisms that lead to these deleterious effects on ARTs are still unclear, so more studies are needed to identify the effects of SDF on ARTs.
